# A Longitudinal HR-pQCT Study of Alendronate Treatment in Postmenopausal Women With Low Bone Density: Relations Among Density, Cortical and Trabecular Microarchitecture, Biomechanics, and Bone Turnover

**DOI:** 10.1002/jbmr.157

**Published:** 2010-06-18

**Authors:** Andrew J Burghardt, Galateia J Kazakia, Miki Sode, Anne E de Papp, Thomas M Link, Sharmila Majumdar

**Affiliations:** 1 Musculoskeletal Quantitative Imaging Research Group, Department of Radiology and Biomedical Imaging, University of California San Francisco, San Francisco, CA, USA; 2 Joint Graduate Group in Bioengineering, University of California San Francisco and Berkeley, San Francisco, CA, USA; 3 Merck & Co., Inc. Whitehouse Station, NJ, USA

**Keywords:** HR-pQCT, MICRO–COMPUTED TOMOGRAPHY, BONE STRUCTURE, BIOMECHANICS, OSTEOPOROSIS, ALENDRONATE

## Abstract

The goal of this study was to characterize longitudinal changes in bone microarchitecture and function in women treated with an established antifracture therapeutic. In this double-blind, placebo-controlled pilot study, 53 early postmenopausal women with low bone density (age = 56 ± 4 years; femoral neck *T*-score = −1.5 ± 0.6) were monitored by high-resolution peripheral quantitative computed tomography (HR-pQCT) for 24 months following randomization to alendronate (ALN) or placebo (PBO) treatment groups. Subjects underwent annual HR-pQCT imaging of the distal radius and tibia, dual-energy X-ray absorptiometry (DXA), and determination of biochemical markers of bone turnover (BSAP and uNTx). In addition to bone density and microarchitecture assessment, regional analysis, cortical porosity quantification, and micro-finite-element analysis were performed. After 24 months of treatment, at the distal tibia but not the radius, HR-pQCT measures showed significant improvements over baseline in the ALN group, particularly densitometric measures in the cortical and trabecular compartments and endocortical geometry (cortical thickness and area, medullary area) (*p* < .05). Cortical volumetric bone mineral density (vBMD) in the tibia alone showed a significant difference between treatment groups after 24 months (*p* < .05); however, regionally, significant differences in Tb.vBMD, Tb.N, and Ct.Th were found for the lateral quadrant of the radius (*p* < .05). Spearman correlation analysis revealed that the biomechanical response to ALN in the radius and tibia was specifically associated with changes in trabecular microarchitecture (|ρ| = 0.51 to 0.80, *p* < .05), whereas PBO progression of bone loss was associated with a broad range of changes in density, geometry, and microarchitecture (|ρ| = 0.56 to 0.89, *p* < .05). Baseline cortical geometry and porosity measures best predicted ALN-induced change in biomechanics at both sites (ρ > 0.48, *p* < .05). These findings suggest a more pronounced response to ALN in the tibia than in the radius, driven by trabecular and endocortical changes. © 2010 American Society for Bone and Mineral Research.

## Introduction

Osteoporosis is a condition characterized by loss of bone mass and deterioration of microarchitecture, and it manifests clinically as an increased risk of fracture.(1) Currently, determination of fracture risk is based primarily on bone mineral density (BMD) values obtained through areal or volumetric X-ray-based imaging techniques. While BMD has been shown to have utility in predicting bone strength, it does not entirely determine fracture risk(2,3) or adequately assess the full impact of therapeutic interventions.(4,5) For these reasons, much interest currently is directed at the investigation of other factors associated with bone mechanical competence and therapeutic response, including whole-bone geometry, cortical and trabecular microarchitecture, and tissue composition.

Analysis of bone structure is critical to understanding bone mechanics, assessing fracture risk, and evaluating responses to disease, age, and therapy. Improved predictions of biomechanical properties have been found as a result of including measures of trabecular bone structure in statistical regressions.(6,7) Trabecular bone structure is also critical in the evaluation of therapeutic interventions, enabling researchers to explain a greater proportion of the effect of drugs on fracture risk than BMD alone.(8,9) Therefore, structural analysis is a particularly important aspect of bone quality assessment.

High-resolution peripheral quantitative computed tomography (HR-pQCT) has been introduced recently as a noninvasive method for in vivo 3D characterization of bone microarchitecture in humans. Similar to traditional quantitative computed tomography (QCT), HR-pQCT provides the ability to quantitatively assess volumetric bone mineral density (vBMD) in a compartmental fashion. Additionally, it permits quantification of the geometric, microarchitectural, and mechanical features of human cortical and trabecular bone in the appendicular skeleton (distal radius and tibia). This imaging technique has been applied in a number of cross-sectional studies to characterize aging effects,(1,10–14) fracture discrimination,(15–18) and various pathologies.(19–21) The potential of HR-pQCT to provide longitudinal in vivo assessment of bone quality with respect to therapeutic efficacy is of particular interest, although, to date, few longitudinal results have been reported.(19,22)

In this study, HR-pQCT has been used to conduct a 2-year longitudinal evaluation of the response to an established antiresorptive therapeutic agent (alendronate) in the distal radius and tibia of early postmenopausal women with low bone density. Previously, comparisons between baseline HR-pQCT and high-resolution magnetic resonance imaging (MRI) microarchitectural measures were reported by Kazakia and colleagues for this cohort.(23) Our aims for the longitudinal component of this study were (1) to characterize the densitometric, geometric, microarchitectural, and biomechanical cortical and trabecular bone changes in subjects treated for 24 months with alendronate compared with placebo, (2) to investigate the relationship between bone density and structure and functional changes, as measured by biochemical markers of bone turnover and estimates of bone strength determined by micro-finite-element analysis (µFEA) following treatment with alendronate, and (3) to investigate the relationship between baseline bone density and structure phenotypes and the functional response to alendronate therapy.

## Materials and Methods

### Subjects

A longitudinal pilot imaging study comparing the effects of alendronate with those of placebo (calcium and vitamin D) on bone microarchitecture was conducted in a total of 53 community-dwelling, ambulatory, postmenopausal women defined as osteopenic by World Health Organization (WHO) criteria.(24) A detailed baseline characterization of this study group has been reported elsewhere.(23) The women were between the ages of 45 and 65 years, with a mean age of 55.6 years (Table 1). The women were postmenopausal for at least 1 but not more than 6 years, with a mean duration of 31 months postmenopause. The subjects had low BMD levels (*T*-score range −1.1 to −2.5) by dual-energy X-ray absorptiometry (DXA) at either the lumbar spine or the total proximal femur, trochanter, or neck regions of interest. A total of 53 women were randomized 1:1—26 in the treatment group [70 mg of alendronate once weekly and daily 2800 IU of vitamin D_3_ and OSCal + D (1000 mg of calcium + 400 IU of vitamin D_3_)] and 27 in the placebo group [daily 2800 IU of vitamin D_3_ and OSCal + D (1000 mg of calcium + 400 IU of vitamin D_3_)]. The study size was based on power calculations of previous longitudinal µMRI data indicating a required sample size of 23 per group in order to detect a treatment difference of 5% (power = 0.95, α = 0.05) in trabecular number at the distal radius.(25) Exclusion criteria included a history of fracture after age 50, history of or evidence for metabolic bone disease other than postmenopausal bone loss, treatment within the previous year with any compound known to influence bone turnover, and estrogen use within the past 6 months. Other exclusion criteria related to indications for specific imaging techniques included in the full study protocol (DXA, HR-pQCT, and MRI) were subject weight greater than 250 lb (113.4 kg), the presence of a pacemaker, and claustrophobia. The UCSF Committee of Human Research approved the study protocol, and all patients gave written informed consent prior to participation.

**Table 1:** Study Participant Characteristics^a^

	Baseline		24 months	
		
	PBO (*n* = 27)	ALN (*n* = 26)	PBO (*n* = 13)	ALN (*n* = 20)
Age (years)	55.4 ± 3.3	55.8 ± 3.9	56.4 ± 2.4	56.1 ± 4.1
Total-hip *T*-score	−0.8 ± 0.6	−0.8 ± 0.6	−0.8 ± 0.5	−0.7 ± 0.7
Lumbar spine *T*-score	−1.0 ± 0.7	−1.2 ± 0.7	−1.1 ± 0.7	−1.1 ± 0.7
Months since menopause	31.5 ± 17.8	30.5 ± 17.4	33.5 ± 18.6	32.0 ± 16.6

a No statistically significant differences between PBO and ALN at baseline or 24 months.

### DXA measurements

Bone densitometry data were acquired using DXA at the lumbar spine, proximal femur (neck, trochanter, and total proximal femur), and distal radius (ultradistal and distal one-third radius). At baseline, 42 subjects were scanned with the QDR 4500 (Hologic, Inc., Bedford, MA, USA) and 11 subjects using the Lunar Prodigy (GE Healthcare, Waukesha, WI, USA). Follow-up measurements were obtained on the same scanner for each subject. *T*-scores were calculated at all sites based on the standard Third National Health and Nutrition Examination Survey (NHANES III) database.

### Biochemical markers of bone turnover

Laboratory parameters used to evaluate changes in bone turnover included urinary *N*-telopeptide of type I human collagen (uNTx; Osteomark NTx EIA, Inverness Medical Professional Diagnostics, Princeton, NJ, USA) corrected for creatinine (Cr; Roche Diagnostics, Indianapolis, IN, USA) to assess the rate of bone resorption and serum bone-specific alkaline phosphatase (BSAP; MicroVue BAP EIA, Quidel Corporation, San Diego, CA, USA) to assess the rate of bone formation. Stored samples were analyzed by a central laboratory (Pacific Biometrics, Seattle, WA, USA) in batches by patient at the end of the study. All biochemical markers of bone turnover were obtained at baseline, 12 months, and 24 months while subjects were fasting. uNTx was measured from second-morning-void urine samples.

### HR-pQCT imaging

**Fig. 1:**
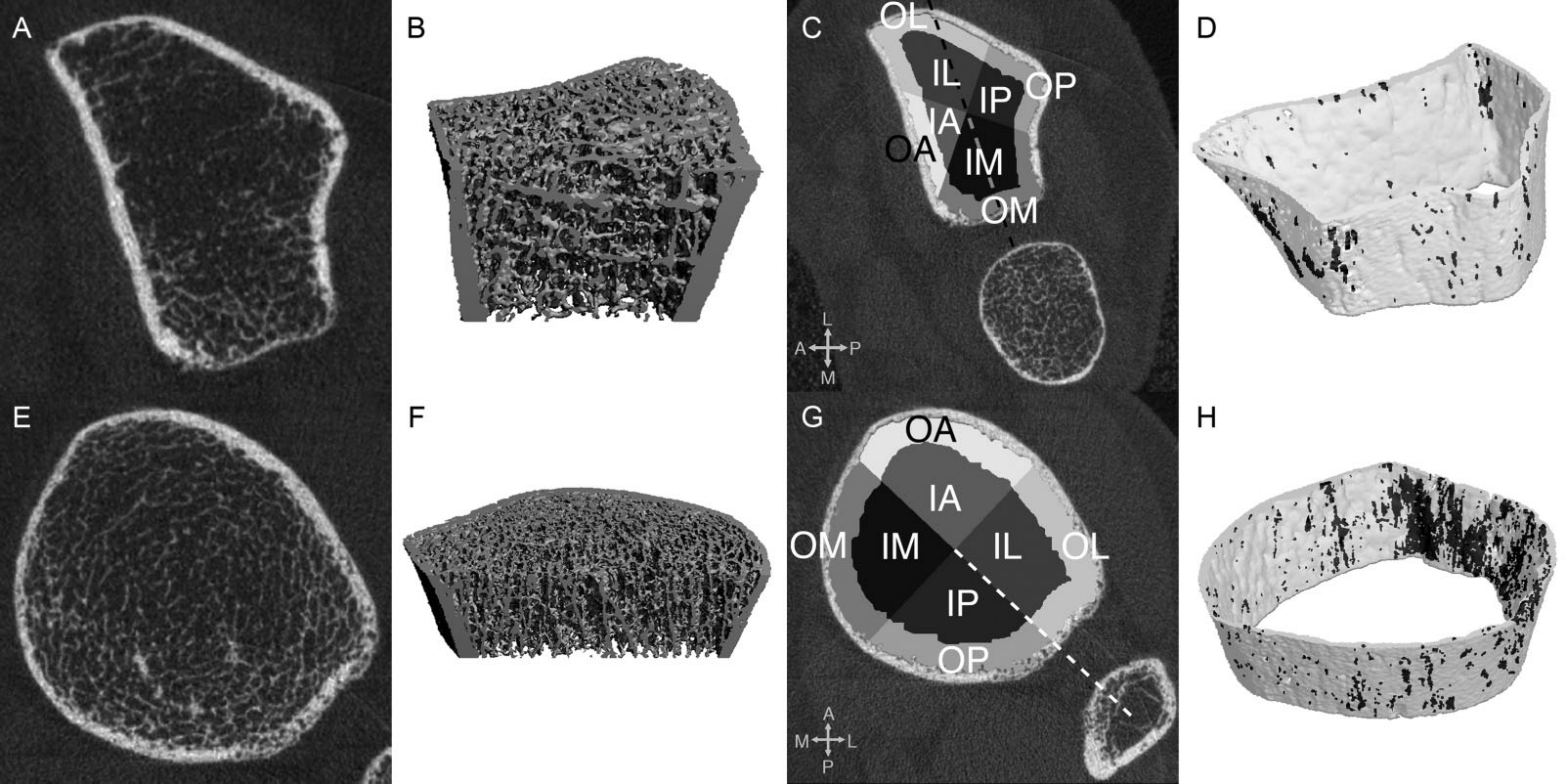
Representative 2D and 3D images of the radius (*A*, *B*) and tibia (*E*, *F*) of a study participant. Definitions for region-based analyses and the segmentation of intracortical porosity are also shown for the radius (*C*, *D*) and tibia (*G*, *H*).

All subjects were imaged in a clinical HR-pQCT system (XtremeCT, Scanco Medical AG, Brüttisellen, Switzerland) using the manufacturer's standard in vivo protocol described in previous patient studies.(15–17) The subject's forearm/ankle was immobilized in a carbon fiber cast that was fixed within the gantry of the scanner. A single dorsal-palmar projection image of the distal radius/tibia was acquired to define the tomographic scan region. This region spanned 9.02 mm in length (110 slices) and was fixed starting at 9.5 and 22.5 mm (for the radius and tibia, respectively) proximal from the middle joint line and extending proximally. For tomography, 750 projections were acquired over 180 degrees with a 100-ms integration time at each angular position. The 12.6-cm field of view (FOV) was reconstructed across a 1536 × 1536 matrix using a modified Feldkamp algorithm, yielding 82-µm voxels.(26) Total scan time was 2.8 minutes, with an effective dose of approximately 4.2 µSv for each site. Representative cross-sectional and 3D images are shown in Fig. 1.

### HR-pQCT analysis

#### Image registration

All longitudinal analyses were constrained to a common volume of interest (VOI) between baseline and 12- and 24-month measurements. This common region was identified using software provided by the manufacturer. The method computes periosteal cross-sectional area on a slice-by-slice basis for each time point and then determines an optimal offset between scans using a cross-correlation approach. As a result, the VOI generally was less than the full 110 slices acquired (average 103 slices). All subsequent analyses of HR-pQCT data were applied to the common VOI determined in this fashion.

#### Standard analysis

HR-pQCT images were evaluated using the standard clinical evaluation protocol, adapted from methods developed for a previous pQCT device.(27,28) First, the periosteal perimeter was identified using a semiautomated edge-finding algorithm that produces a closed contour around the periosteal surface. The total volumetric bone mineral density (vBMD) was calculated from the volume contained within this contour. The cortical compartment VOI was segmented using a 3D Gaussian operator to smooth out the trabecular structure followed by a fixed threshold (16% of the positive integer space) to separate mineralized cortex from background. Cortical vBMD (Ct.vBMD) was calculated as the mean density of voxels in the segmented cortical VOI. Cortical thickness (Ct.Th) was calculated using an annular model approximation: cortical area (Ct.Ar) divided by the periosteal perimeter. The trabecular compartment was identified by subtraction of a mask of the cortical compartment from a mask of the full region encompassed by the periosteal contour. The trabecular bone volume to total volume ratio (BV/TV) was derived from the BMD of the trabecular VOI (Tb.vBMD) and making the assumption that compact bone has a matrix mineral density of 1200 mg hydroxyapatite (HA)/cm^3^, whereas the marrow background is equivalent to 0 mg HA/cm^3^. Extraction of the trabecular structure was performed as follows: A Laplace-Hamming filter—which effectively smoothes the image and enhances edges—was applied to the original grayscale data, and a fixed global threshold (40% of the positive integer space) was applied to discretize the bone and background phases.(29) From the binary image, Tb.N was calculated directly by computing the mean of the 3D distance between trabecular midaxes.(30,31) The standard deviation of those distances (Tb.1/N.SD) also was calculated to represent the spatial heterogeneity of the microarchitecture. Based on the densitometric BV/TV and direct Tb.N, Tb.Th and Tb.Sp were derived using traditional plate model assumptions.(32)

#### Subregional analysis

In addition to the standard patient-style evaluation, which results in a global average value for the entire volume, the following process for defining subregions was performed automatically using a method described recently by Sode and colleagues.(33) The trabecular compartment was divided into two concentric regions (inner and outer subregions), where the area of the inner subregion was 60% of the entire trabecular region. The volume was divided further into axial quadrants. In the lower limb, a line connecting the axial centroids of the tibia and fibula and its orthogonal complement defined approximately mediolateral/anteroposterior quadrants. In the forearm, the major and minor axes of the axial cross section of the radius defined mediolateral/anteroposterior quadrants. A total of 8 subregions resulted, as shown in Fig. 1*C, G*. Each subregion was denoted by a two-letter acronym based on location: I or O for inner or outer subregions, respectively, and M, P, L, or A for medial, posterior, lateral, or anterior, respectively (Fig. 1*C, G*). Mean cortical and trabecular densitometric and microarchitectural indices were computed for each individual subregion.

#### Extended cortical bone analysis

As reported previously, the default cortical bone analysis performs poorly for subjects with very thin or highly porous cortices.(23,34) Accordingly, a fully automated cortical compartment segmentation technique adapted from the method described previously by Buie and colleagues was applied.(34,35) To exclusively segment the intracortical porosity volume, a novel algorithm based on 2D component labeling and a 3D region-growing process was applied.(36) True intracortical porosity was estimated initially to be all void voxels unconnected to the background in each 2D axial slice. Next, a region-growing process additionally included void voxels connected along the *z* axis (SI direction) to the initial pore voxel (Fig. 1*D, H*). Based on this segmentation of the intracortical pore volume (Ct.PoV), intracortical porosity (Ct.Po) was defined as a normalized volumetric index according to Eq. (1):


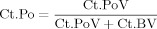
(1)

where Ct.BV is the mineralized cortical bone volume. Based on this segmentation, a direct 3D calculation(30) of cortical thickness (Ct.Th*) was performed on the composite segmentations of the mineralized cortex and porosity, thereby disregarding intracortical pore surfaces in the local distance calculations. This index represents a direct 3D measure of endosteal-periosteal distance. Previous reproducibility measurements in 27 postmenopausal women indicated the least significant change for Ct.Po to be 0.57% (RMSCV% = 11.7%) for the distal radius and 0.92% (RMSCV% = 3.9%) for the distal tibia.

#### µFE analysis

Linear micro-finite-element analysis (µFEA) was applied to calculate apparent biomechanical properties under uniaxial compression. Homogeneous mechanical properties were assumed for all bone elements. The binary image data set was converted to a mesh of isotropic brick elements using a voxel conversion technique,(37) and each element was assigned an elastic modulus of 10 GPa(38) and a Poisson's ratio of 0.3.(39) Cortical and trabecular bone elements were labeled as different materials with identical material properties to facilitate calculation of compartmental load distribution. A uniaxial compression test in the axial direction (superoinferior) was performed with an applied strain of 1%. An iterative solver (Scanco FE Software, Version 1.12, Scanco Medical) was used to compute reaction forces at the proximal and distal ends of the scan region for the prescribed displacements. The model computations were performed at the UCSF/QB3 Shared Computing Facility—a mixed-architecture Linux HPC grid consisting of 1900 processor nodes. No models were excluded owing to convergence limitations.

For each model, stiffness *K*, apparent modulus *E*, and the load fraction for the cortical compartment (Ct.LF) at the distal boundary were calculated. Furthermore, failure load *F* was estimated using methods previously described by Pistoia and colleagues.(40) Differential biomechanical indices related to cortical porosity also were calculated by running a second model for each specimen with intracortical pore spaces digitally occluded.(36) Specifically, the deficit in stiffness Δ*K*_PO_, apparent modulus Δ*E*_PO_, and failure load Δ*F*_PO_ owing to the resolvable intracortical porosity were calculated as the difference in the respective parameter between the occluded and original models and normalized by the original. These indices were reported as a percent. The fractional load (ΔCt.LF_PO_) shifted from the cortex to the trabecular bone was calculated simply as the difference between occluded and original Ct.LF.

### Statistical analysis

The statistical analysis was performed on a per-protocol basis. The change from baseline at 12 and 24 months for all parameters was expressed as the percent difference from baseline in alendronate-treated (ALN) and placebo-treated (PBO) groups. The statistical significance of the change with respect to baseline as well as between treatment groups was assessed using a two-way analysis of variance with repeated measures on time (RMANOVA). Nonparametric Spearman correlations were used to characterize the response to ALN or PBO treatment. For the purposes of this analysis, response was considered to be absolute change in the biomarkers uNTx/Cr and BSAP as well as the percent change in µFEA-derived biomechanical indices following 24 months of treatment. Specifically, the following relationships were investigated: (1) the association between changes in BMD, geometry, and microarchitecture and response to ALN and PBO treatment at 24 months and (2) whether baseline bone turnover, BMD, microarchitecture, or biomechanics predicted the subsequent response to ALN treatment after 24 months. A significance level of *p* < .05 was considered statistically significant. All statistical tests were performed using JMP (Version 7.0, SAS Institute, Inc., Cary NC, USA).

## Results

### Final subject disposition

The 24-month subject attrition rate was 23% for the ALN group (*n* = 20) and 52% for the PBO group (*n* = 13). One PBO-treated subject was removed by the principal clinical investigator (blinded to treatment) owing to a large decrease in BMD after 12 months. The remaining dropouts were due to elective withdrawals (PBO = 6, ALN = 3), adverse events (PBO = 3, ALN = 2), nonadherence (PBO = 2, ALN = 1), or unrelated health issues (PBO = 1, ALN = 1). For the subjects who completed the full 24-month study, there were no statistically significant differences in the primary screening inclusion measures (baseline age, months since menopause, total-hip *T*-score, and total-spine *T*-score) between the ALN and PBO groups (Table 1).

### Treatment effects on turnover, density, geometry, microarchitecture, and strength

#### Biochemical markers of bone turnover and DXA

There was a significant reduction in both BSAP and uNTx between baseline and follow-up visits for both the PBO and ALN groups (*p* < .05). A significantly greater reduction in bone turnover was seen with ALN than with PBO after 12 and 24 months (*p* < .0001).

Lumbar spine DXA areal BMD (aBMD) values increased in the treated group (*p* < .0001), whereas the PBO group showed maintenance at 12 months and a small decline at 24 months. Treatment effects in the spine were significant at both 12 and 24 months (*p* < .0001). Increases in aBMD were greater in the lumbar spine compared with the total hip, although the ALN group showed significant changes from baseline (*p* < .001) and compared with PBO (*p* < .05) at both sites. Both total and ultradistal forearm densities were maintained in the ALN group and dropped significantly in PBO-treated subjects (*p* < .05; except at 12 months for the ultradistal forearm). Significant treatment effects were observed for the ultradistal forearm (*p* < .05) but not for the total forearm.

#### Longitudinal and treatment effects on HR-pQCT parameters

**Fig. 2:**
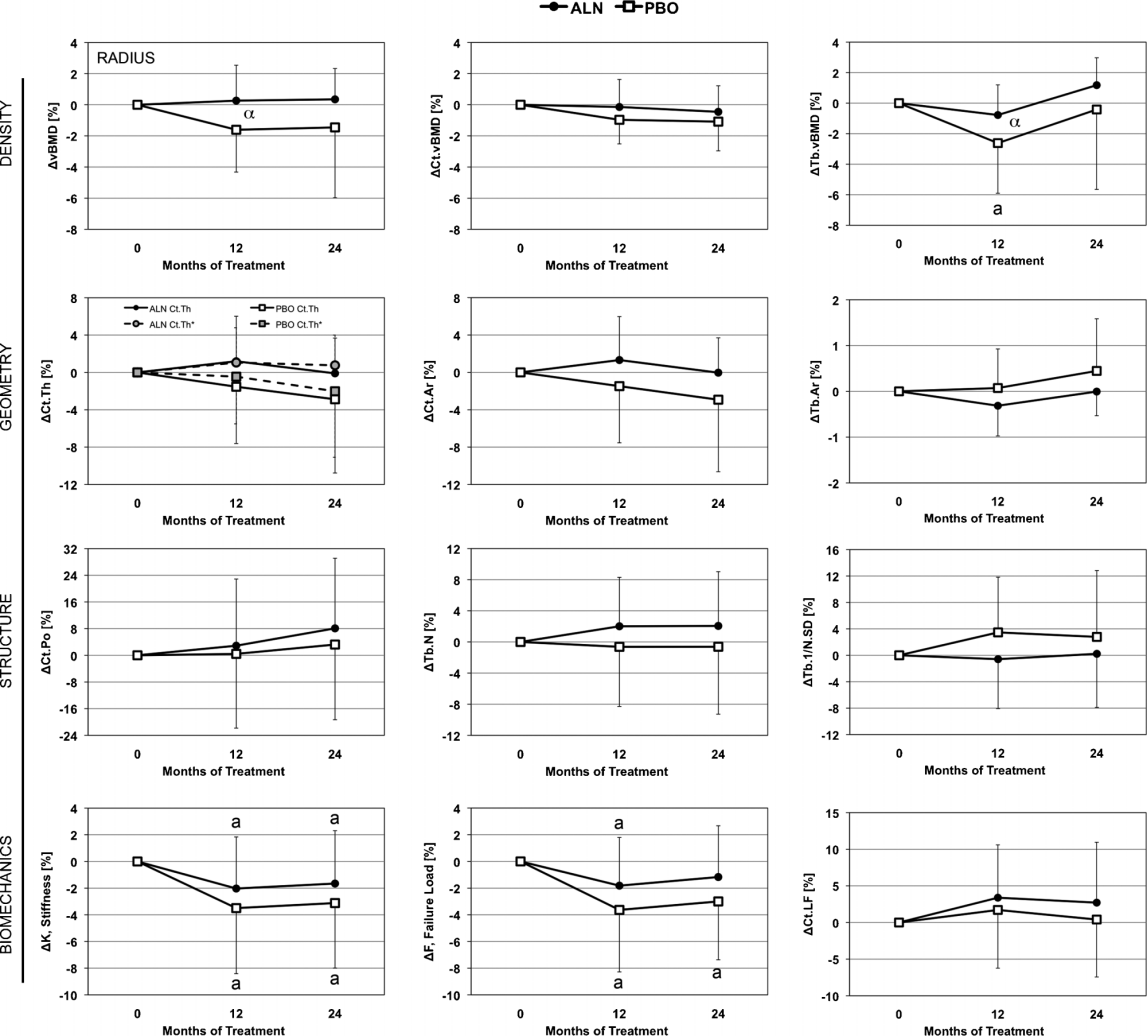
Time-series line plots of the percent change in *distal radius* HR-pQCT densitometric, geometric, microarchitectural and biomechanical indices for ALN-treated subjects (*black circle*) and PBO controls (*white box*). Cortical thickness is presented as calculated by the standard analysis (*solid line*) and from the direct 3D endosteal to periosteal distance after removing intracortical pore surfaces (*dashed line*). (^a^*p* < .05; ^b^*p* < .01; ^c^*p* < .001; ^d^*p* < .0001 versus baseline; ^α^*p* < .05, ^β^*p* < .01, ^χ^*p* < .001, ^δ^*p* < .0001 ALN versus PBO).

**Fig. 3:**
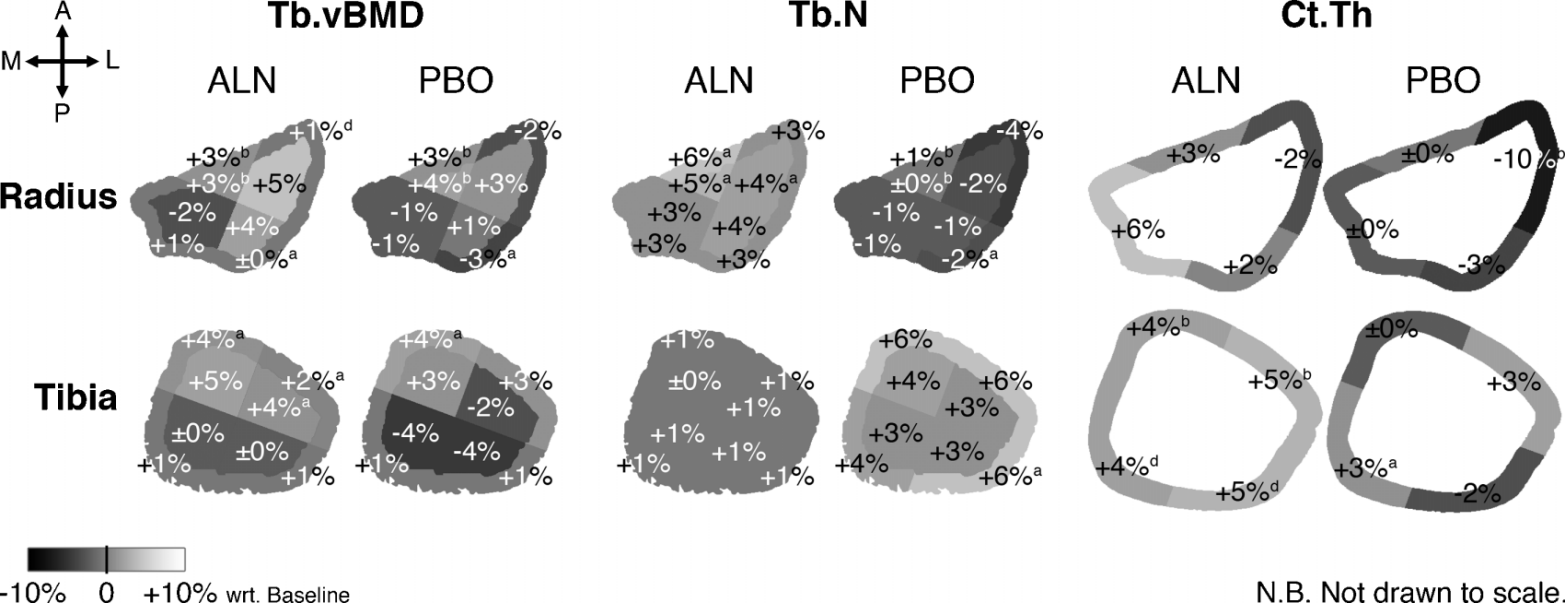
Mean percent differences in Tb.vBMD, Tb.N, and Ct.Th at each region of the distal radius and tibia at 24 months compared with baseline for each group. ^a^*p* < .05; ^b^*p* < .01; ^c^*p* < .001; ^d^*p* < .0001; otherwise, not significant.

**Fig. 4:**
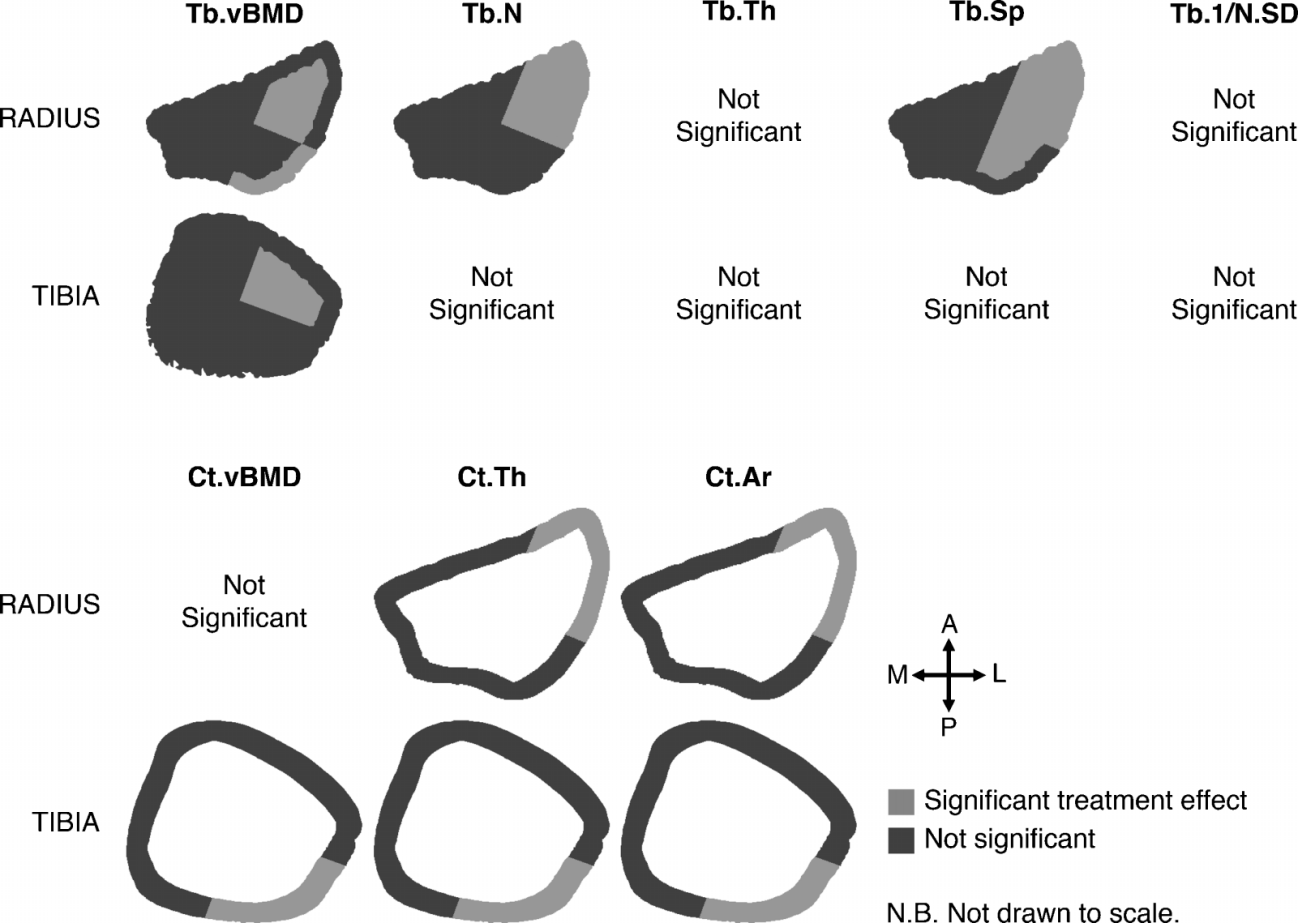
A graphic table summarizing the regions where a significant treatment effect was observed for trabecular and cortical parameters after 24 months (highlighted in light gray), determined by MANOVA with time as a repeated measure with α = 0.05.

In the distal radius (Fig. 2), HR-pQCT density measures tended to decrease after 12 and 24 months in the PBO-treated cohort. This decrease was statistically significant only for Tb.vBMD after 12 months (*p* < .05). In contrast, ALN-treated subjects tended to maintain bone density, with significant treatment differences for total vBMD and Tb.vBMD at 12 months (*p* < .05). Loss of Tb.vBMD was particularly pronounced in the outer posterior region for PBO subjects (−3%, *p* < .05; Fig. 3), which, by comparison, was preserved in ALN-treated subjects (*p* < .05; Fig. 4). Additionally, a significant treatment effect for Tb.vBMD was seen in the inner lateral region of the radius (*p* < .05).

No significant longitudinal or treatment-wise effects were seen for the global geometry in the distal radius, although cortical thickness and area tended to decrease whereas medullary area tended to increase in PBO subjects. This was marked by a significant decrease (−10%, *p* < .05) in Ct.Th and Ct.Ar of the lateral cortical region that was maintained with ALN treatment (*p* < .05). Similarly, no significant longitudinal or treatment effects were seen for global trabecular microarchitecture measures in the radius; however, at 24 months, significant treatment-wise differences were found for Tb.N of the lateral regions (*p* < .05), which decreased in PBO subjects (−2% to 4%, not significant with respect to baseline) and increased with ALN (+3% to 4%, not significant with respect to baseline).

Finite-element estimates of bone strength at the distal radius decreased significantly for both PBO and ALN cohorts at 12 and 24 months with respect to baseline (*p* < .05), with the exception of apparent modulus and failure load at 24 months for ALN. While the loss of bone strength tended to be less for the ALN-treated cohort, the treatment effect did not reach statistical significance.

**Fig. 5:**
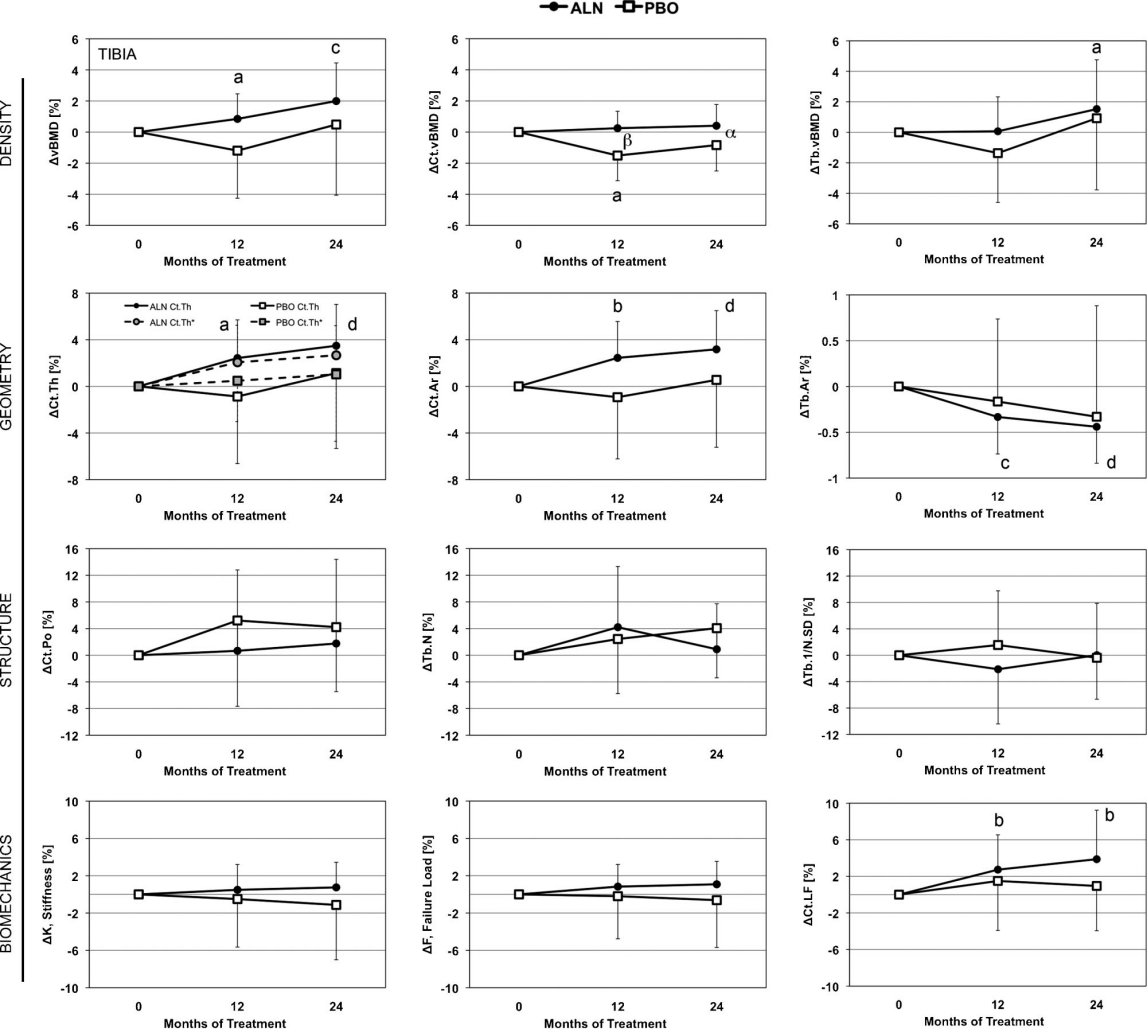
Time-series line plots of the percent change in *distal tibia* HR-pQCT densitometric, geometric, microarchitectural, and biomechanical indices for ALN-treated subjects (*black circle*) and PBO controls (*white box*). Cortical thickness is presented as calculated by the standard analysis (*solid line*) and from the direct 3D endosteal to periosteal distance after removing intracortical pore surfaces (*dashed line*). ^a^*p* < .05, ^b^*p* < .01, ^c^*p* < .001, ^d^*p* < .0001 versus baseline; ^α^*p* < .05, ^β^*p* 0.01, ^χ^*p* < .001, ^δ^*p* < .0001 ALN versus PBO.

In the distal tibia (Fig. 5), total vBMD increased significantly with ALN treatment after 12 and 24 months (*p* < .05 and *p* < .001, respectively). Cortical density decreased significantly at 12 but not 24 months (*p* < .05), whereas ALN significantly maintained Ct.vBMD compared with PBO at 12 and 24 months (*p* < .05 and *p* < .01, respectively). While global trabecular vBMD increased significantly for ALN-treated subjects (*p* < .05), a significant treatment effect was found only for the inner lateral region (*p* < .05), which increased by +4% in the ALN group (*p* < .05 with respect to baseline) and decreased by −2% in the control (not significant with respect to baseline).

Tibial geometry was altered significantly at all time points by ALN. In particular, both the areal estimate and the direct 3D measure of cortical thickness increased by 3% to 4% at 24 months (*p* < .0001 for both). Similarly, cortical area increased and medullary area decreased significantly at 24 months (*p* < .0001 for both). While the global geometric changes did not differ significantly between treatment cohorts, significant treatment effects on Ct.Th and Ct.Ar between ALN and PBO were found for the lateral cortical quadrant at 24 months (*p* < .05). Cortical and trabecular microarchitecture alterations were not detected globally nor within any subregion, and no difference was observed between ALN and PBO groups.

No longitudinal or treatment-wise effects were found for µFEA estimates of bone strength at the distal tibia. However, stiffness and failure load tended to increase with ALN and decrease with PBO. The load fraction supported by the cortex was significantly increased following 12 and 24 months of ALN (*p* < .01).

### Bone changes associated with the functional response to treatment

The Spearman rank correlation coefficient for the 24-month change in each index with respect to the 24-month change in biochemical markers of bone turnover and µFEA-derived axial stiffness *K* and cortical load fraction (Ct.LF) are presented in Tables 2 and 3 for the distal radius and tibia, respectively.

**Table 2:** Spearman Correlations for 24-Month Percent Changes in *Radius* Measures for PBO and ALN^a^

	PBO (N = 13)				ALN (N = 20)			
		
Parameter %Δ	ΔBSAP	ΔuNTx	%Δ*K*	%ΔCt.LF	ΔBSAP	ΔuNTx	%Δ*K*	%ΔCt.LF
DXA								
ΔTotal-spine aBMD	0.19	0.07	0.05	0.46	−0.25	0.27	−0.06	0.17
ΔTotal-femur aBMD	0.12	0.01	0.25	0.25	−0.65^**^	−0.09	−0.14	0.28
ΔTotal-radius aBMD	0.75^**^	0.49	−0.05	0.40	0.04	0.46^*^	0.12	0.10
ΔUltradistal radius aBMD	0.45	0.14	−0.05	0.62^*^	0.07	0.54^*^	0.15	0.20
HR-pQCT density								
ΔvBMD	0.05	−0.34	−0.09	0.71^**^	−0.26	0.19	−0.05	0.36
ΔCt.vBMD	−0.23	−0.25	0.37	0.22	−0.24	−0.10	0.07	−0.08
ΔTb.vBMD	0.32	−0.14	−0.41	0.76^**^	0.06	0.37	−0.32	0.33
HR-pQCT geometry								
ΔCt.Th^*^	0.03	−0.36	0.01	0.64^*^	−0.27	−0.07	0.20	0.29
ΔCt.Ar	−0.04	−0.43	0.04	0.62^*^	−0.36	0.20	0.15	0.32
ΔTb.Ar	−0.07	0.37	−0.10	−0.54	0.29	−0.16	−0.16	−0.37
HR-pQCT structure								
ΔCt.PoV	−0.12	−0.28	0.75^**^	−0.12	−0.09	−0.21	0.02	0.05
ΔCt.Po (%)	−0.16	−0.25	0.77^**^	−0.17	−0.08	−0.21	0.05	−0.01
ΔTb.N	−0.12	−0.21	−0.59^*^	0.89^****^	−0.19	0.35	−0.80^****^	0.64^**^
ΔTb.Th	0.37	0.04	0.45	−0.49	0.16	−0.31	0.78^****^	−0.60^**^
ΔTb.Sp	0.02	0.19	0.59^*^	−0.92^****^	0.19	−0.32	0.79^****^	−0.60^**^
ΔTb.1/N.SD	0.01	−0.04	0.47	−0.65^*^	0.02	−0.33	0.56^*^	−0.51^*^
HR-pQCT porosity biomechanics								
ΔΔ*K*_PO_	−0.20	−0.27	0.42	0.23	−0.26	−0.19	−0.21	0.18
ΔΔ*E*_PO_	−0.23	−0.32	0.38	0.25	−0.29	−0.16	−0.23	0.21
ΔΔ*F*_PO_	−0.62^*^	−0.26	0.35	−0.21	−0.14	0.12	−0.03	0.10
ΔΔCt.LF_PO_	−0.43	−0.23	0.85^***^	−0.46	0.04	−0.36	0.13	−0.44

a Bold indicates statistically significant correlations with ^*^p < .05, ^**^p < .01, ^***^p < .001, ^****^p < .0001

**Table 3:** Spearman Correlations for 24-Month Percent Change in *Tibia* Measures for PBO and ALN^a^

	PBO (n = 13)				ALN (n = 20)			
		
Parameter %Δ	ΔBSAP	ΔuNTx	%Δ*K*	%ΔCt.LF	ΔBSAP	ΔuNTx	%Δ*K*	%ΔCt.LF
DXA								
ΔTotal-spine aBMD	0.19	0.07	0.38	−0.03	−0.25	0.27	0.18	0.26
ΔTotal-femur aBMD	0.12	0.01	−0.06	0.36	−0.65^**^	−0.09	0.16	0.20
ΔTotal-radius aBMD	0.75^**^	0.49	−0.44	0.51	0.04	0.46	0.26	−0.42
ΔUltradistal radius aBMD	0.45	0.14	0.29	−0.04	0.07	0.54^*^	−0.02	−0.35
HR-pQCT density								
ΔvBMD	−0.05	−0.25	0.59^*^	0.11	0.02	0.27	0.19	0.37
ΔCt.vBMD	0.03	−0.01	0.58^*^	−0.27	0.23	0.28	0.22	0.08
ΔTb.vBMD	0.04	−0.23	0.05	−0.10	−0.14	0.10	0.10	0.56^**^
HR-pQCT geometry								
ΔCt.Th^*^	−0.17	−0.43	0.76^**^	0.10	−0.24	−0.09	0.15	0.36
ΔCt.Ar	−0.06	−0.35	0.64^*^	0.21	0.12	0.26	0.26	0.16
ΔTb.Ar	0.20	0.31	−0.76^**^	−0.05	−0.03	−0.12	−0.50^*^	0.01
HR-pQCT structure								
ΔCt.PoV	−0.03	−0.25	0.68^*^	0.16	−0.18	−0.41	−0.15	−0.06
ΔCt.Po	−0.20	−0.19	0.78^**^	−0.11	−0.08	−0.39	−0.14	−0.14
ΔTb.N	0.51	0.53	−0.71^*^	0.63^*^	0.00	0.04	−0.37	0.63^**^
ΔTb.Th	−0.52	−0.48	0.69^*^	−0.78^**^	−0.08	0.01	0.61^**^	−0.43
ΔTb.Sp	−0.45	−0.54	0.69^*^	−0.64^*^	0.03	−0.06	0.35	−0.63^**^
ΔTb.1/N.SD	−0.30	−0.46	0.07	−0.21	−0.03	0.11	0.50^*^	−0.40
HR-pQCT porosity biomechanics								
ΔΔ*K*_PO_	0.15	−0.06	0.48	0.15	−0.19	−0.43	−0.52^*^	0.13
ΔΔ*E*_PO_	0.12	−0.06	0.54	0.12	−0.20	−0.43	−0.51^*^	0.11
ΔΔ*F*_PO_	0.08	−0.09	0.38	0.23	−0.03	−0.49^*^	−0.32	−0.07
ΔΔCt.LF_PO_	−0.22	0.15	0.50	−0.19	0.11	−0.46^*^	−0.10	−0.24

a Bold indicates statistically significant correlations with ^*^p < .05, ^**^p < .01, ^***^p < .001, ^****^p < .0001.

**Fig. 6:**
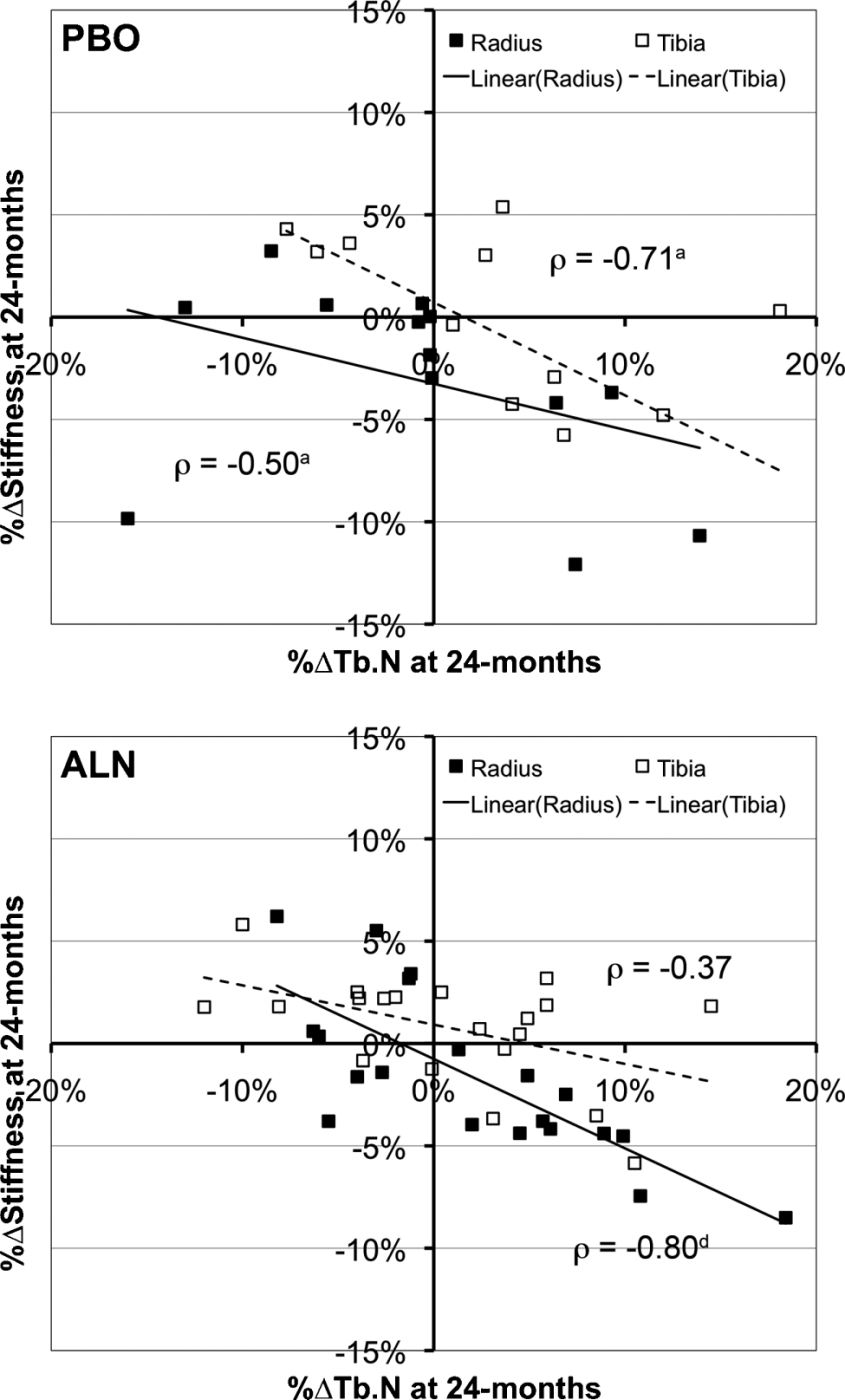
Scatter plot for the percent change in trabecular number against the change in axial stiffness computed by µFEA following 24 months of treatment with placebo (*top*) or alendronate (*bottom*). Both cohorts exhibited moderate to strong Spearman correlation coefficients. ^a^*p* < .05; ^b^*p* < .01; ^c^*p* < .001; ^d^*p* < 0.0001; otherwise, not significant.

In the distal radius (Table 2), the change in biomarker measures after 24 months generally correlated poorly with the change in HR-pQCT measures in the PBO and ALN groups. However, the change in biomechanical measures at 24 months was found to correlate significantly with a number cortical and trabecular bone changes in PBO-treated subjects. In contrast, the biomechanical response to ALN treatment was specifically associated with changes in trabecular microarchitecture alone (0.51 ≤ |ρ| ≤ 0.80, *p* < .05); the change in Tb.N was strongly negatively correlated with the change in stiffness (Fig. 6), and the changes in Tb.Th and Tb.Sp were equally positively correlated with the change in stiffness.

In the distal tibia (Table 3), similar structure-function relationships were observed. For both the PBO and ALN groups, the changes in biomarker measures were not found to correlate significantly with changes in HR-pQCT measures or density or structure measures. In the PBO cohort, the 24-month change in stiffness significantly correlated with changes in both cortical and trabecular structure. In particular, cortical thickness and porosity were strongly positively correlated with the change in stiffness (ρ > 0.75, *p* < .01), whereas medullary area (Tb.Ar) and Tb.N were strongly negatively correlated with the change in stiffness (ρ < −0.70, *p* < .05). In the ALN group, the biomechanical response in the tibia was more moderately associated with trabecular and endocortical changes (Tb.Ar, Tb.Th, Tb.1/N.SD; 0.50 ≤ |ρ| ≤ 0.61, *p* < .05).

### Baseline predictors of the functional response to ALN

The Spearman rank correlation coefficient for each baseline index with respect to the 24-month changes in biochemical markers of bone turnover and µFEA-derived estimates of bone strength are presented in Tables 4 and 5 for the distal radius and tibia, respectively, for the ALN cohort.

**Table 4:** Spearman Correlations for Baseline *Radius* Measures Against 24-Month Response to ALN^a^

	Biomarkers				Biomechanics			
		
Baseline parameter	BSAP baseline	uNTx baseline	ΔBSAP	ΔuNTx	%Δ*K*	%Δ*E*	%Δ*F*	%ΔCt.LF
Biomarkers								
BSAP	—	0.31^*^	−0.66^***^	−0.31	−0.2	−0.26	−0.34	0.07
uNTx	0.31^*^	—	0.15	−0.77^***^	0.25	0.12	0.24	−0.47^*^
DXA								
Total-spine aBMD	−0.03	0.09	0.34	0.19	0.01	0.03	0.05	−0.05
Total-femur aBMD	−0.07	0.03	0.20	0.22	−0.25	−0.29	−0.17	0.19
Total-radius aBMD	−0.20	−0.31^*^	−0.12	0.30	−0.09	−0.13	−0.16	0.54^*^
Ultradistal radius aBMD	0.04	−0.10	−0.15	0.13	−0.13	−0.22	−0.24	0.49^*^
HR-pQCT density								
vBMD	0.03	0.20	−0.10	−0.27	0.14	−0.06	0.00	0.01
Ct.vBMD	0.13	−0.19	−0.17	−0.11	0.08	−0.02	−0.05	−0.06
Tb.vBMD	−0.14	0.15	0.22	0.04	−0.22	−0.37	−0.32	0.31
HR-pQCT geometry								
Ct.Th*	0.19	0.05	−0.10	−0.24	0.32	0.22	0.26	−0.12
Ct.Ar	0.17	−0.02	−0.12	−0.09	0.16	0.09	0.09	0.17
Tb.Ar	−0.08	−0.19	−0.18	0.18	−0.18	−0.13	−0.18	0.35
HR-pQCT structure								
Ct.PoV	−0.07	0.37^**^	0.25	−0.01	0.35	0.42	0.48^*^	0.04
Ct.Po (%)	−0.12	0.34^*^	0.29	−0.05	0.31	0.37	0.43	0.02
Tb.N	−0.18	0.10	0.32	0.02	−0.06	−0.19	−0.12	0.33
Tb.Th	−0.04	0.16	0.02	−0.05	−0.28	−0.32	−0.31	0.03
Tb.Sp	0.19	−0.11	−0.29	−0.04	0.10	0.23	0.18	−0.37
Tb.1/N.SD	0.18	0.00	−0.27	−0.25	0.36	0.44	0.43	−0.47^*^
HR-pQCT biomechanics								
Stiffness *K*	0.06	0.15	−0.18	−0.12	−0.25	−0.32	−0.30	0.50^*^
Modulus *E*	0.01	0.15	−0.25	−0.38	−0.01	−0.17	−0.13	−0.04
Failure load *F*	0.05	0.12	−0.19	−0.16	−0.30	−0.40	−0.38	0.46^*^
Ct.LF	0.19	0.05	−0.02	−0.34	0.62^**^	0.62^**^	0.64^**^	−0.74^***^
Δ*K*_PO_	−0.08	0.35^*^	0.30	−0.04	0.43	0.50^*^	0.54^*^	−0.03
Δ*E*_PO_	−0.09	0.34^*^	0.35	0.02	0.47^*^	0.55^*^	0.58^*^	−0.06
Δ*F*_PO_	−0.11	0.32^*^	0.26	−0.06	0.36	0.41	0.50^*^	0.03
ΔCt.LF_PO_	−0.17	**0.28**^a^	0.26	0.18	0.08	0.14	0.23	0.35

a Bold indicates statistically significant correlations with ^*^p < .05, ^**^p < .01, ^***^p < .001, ^****^p < .0001.

**Table 5:** Spearman Correlations for Baseline *Tibia* Measures Against 24-Month Response to ALN^a^

	Biomarkers				Biomechanics			
		
Baseline parameter	BSAP baseline	uNTx baseline	ΔBSAP	ΔuNTx	%Δ*K*	%Δ*E*	%Δ*F*	%ΔCt.LF
Biomarkers								
BSAP	—	0.29^*^	−0.72^***^	−0.31	0.12	0.00	0.05	0.49^*^
uNTx	0.29^*^	—	−0.02	−0.76^***^	0.06	0.05	−0.08	−0.15
DXA								
Total-spine aBMD	−0.02	0.10	0.34	0.19	0.19	0.20	0.08	0.06
Total-femur aBMD	−0.02	0.07	0.20	0.22	0.18	0.30	0.06	−0.11
Total-radius aBMD	−0.23	−0.32^*^	−0.12	0.30	0.29	0.21	0.27	0.10
Ultradistal radius aBMD	0.01	−0.13	−0.15	0.13	0.10	0.09	0.07	−0.01
HR-pQCT density								
vBMD	0.02	0.11	0.12	−0.07	0.29	0.52^*^	0.20	−0.62^**^
Ct.vBMD	0.17	−0.24	−0.36	−0.12	0.35	0.50^*^	0.28	−0.27
Tb.vBMD	−0.41^*^	0.01	0.51^*^	0.24	0.15	0.16	0.12	−0.42
HR-pQCT geometry								
Ct.Th*	0.22	0.13	0.05	−0.29	0.19	0.46^*^	0.10	−0.57^**^
Ct.Ar	0.09	0.08	0.12	−0.33	0.21	0.46^*^	0.15	−0.63^**^
Tb.Ar	−0.28^*^	−0.22	−0.10	0.19	−0.18	−0.42	−0.06	0.49^*^
HR-pQCT structure								
Ct.PoV	−0.11	0.26	0.56^*^	0.05	−0.14	−0.15	−0.06	−0.17
Ct.Po (%)	−0.11	0.27	0.46^*^	0.14	−0.16	−0.24	−0.06	0.05
Tb.N	−0.15	−0.08	0.20	0.39	0.18	0.02	0.11	0.17
Tb.Th	−0.26	0.10	0.37	−0.24	−0.27	−0.10	−0.22	−0.39
Tb.Sp	0.17	0.05	−0.22	−0.37	−0.19	−0.03	−0.12	−0.11
Tb.1/N.SD	0.10	0.13	−0.06	−0.41	−0.24	−0.08	−0.07	−0.05
HR-pQCT biomechanics								
Stiffness *K*	−0.20	0.13	0.07	−0.36	0.03	0.21	0.00	−0.55^*^
Modulus *E*	0.06	0.23	0.12	−0.33	0.03	0.33	−0.06	−0.65^**^
Failure load *F*	−0.26	0.00	0.25	−0.19	−0.06	0.08	−0.08	−0.43
Ct.LF	0.41^**^	0.02	−0.17	−0.40	0.17	0.34	0.04	−0.45^*^
Δ*K*_PO_	0.01	0.31^*^	**0.51**^**B**^	0.09	−0.06	−0.09	0.02	−0.04
Δ*E*_PO_	0.01	**0.30**^a^	**0.51**^a^	0.09	−0.06	−0.09	0.02	−0.04
Δ*F*_PO_	−0.04	**0.28**^a^	**0.53**^a^	0.09	−0.05	−0.11	0.01	−0.05
ΔCt.LF_PO_	−0.26	0.19	**0.48**^a^	0.23	−0.24	−0.35	−0.16	0.12

a Bold indicates statistically significant correlations with ^*^p < .05, ^**^p < .01, ^***^p < .001, ^****^p < .0001.

In the distal radius (Table 4), baseline total radius aBMD, Ct.TMD, and all cortical porosity measures were moderately well correlated with baseline uNTx/Cr (*p* < .05). However, no baseline HR-pQCT measures were found to correlate with the 24-month change in the biochemical markers of bone turnover. In contrast, moderate correlations were found between the 24-month change in biomechanical indices and baseline cortical load fraction (Ct.LF) and porosity-related parameters. In particular, the change in the estimated failure load was positively correlated with baseline Ct.LF, cortical pore volume (Ct.PoV), and all estimates of porosity-related mechanical deficits (ρ ≥ 0.50, *p* < .05).

In the distal tibia (Table 5), baseline Tb.vBMD and all porosity-related volumetric and mechanical indices were positively correlated with the 24-month change in BSAP (0.48 ≤ |ρ| ≤ 0.56, *p* < .05). Biomechanical changes (%Δ*E* and %ΔCt.LF) following 24 months of ALN treatment were significantly correlated with baseline vBMD, Ct.vBMD, Ct.Ar, and Ct.Th (*p* < .05) but not with any measure of trabecular or cortical microarchitecture.

## Discussion

This study reports longitudinal cortical and trabecular microarchitectural changes related to antiresorptive therapy measured by HR-pQCT. In general, ALN resulted in maintenance of vBMD in the radius compared with PBO and a longitudinal increase in the tibia, consistent with the observed reduction in resorption measured by biochemical markers of bone turnover (uNTx). In the radius, these findings also were consistent with the observed maintenance in ultradistal aBMD measured by DXA.

The significant longitudinal increase in cortical thickness, area, and load fraction in the distal tibia and corresponding decrease in medullary area in the ALN-treated group are consistent with the filling of endocortical bone resorption cavities. Both the standard areal estimate of Ct.Th and the direct endosteal-periosteal 3D measure were found to increase significantly with respect to baseline in response to alendronate. On average, cortical thickness increased by 30 µm (areal Ct.Th estimate) and 25 µm (Ct.Th*) coincident with a reciprocal decrease in the radius of the medullary compartment (data not shown). This suggests that the increase in cortical thickness cannot be attributed entirely to a decrease in intracortical porosity nor to an increase in cortical matrix mineralization—neither of which should affect Ct.Th*.

In fact, no significant longitudinal changes in Ct.Po were observed. It must be noted, however, that the spatial resolution of HR-pQCT may be insufficient to directly detect longitudinal changes in porosity on this scale. Furthermore, given the small number of subjects and relatively low precision of Ct.Po parameters (RMSCV = 4% to 11%), the study was not sufficiently powered to detect longitudinal changes in porosity. Since previous ex vivo studies using high-resolution micro–computed tomography (µCT) have found significant changes in iliac crest cortical porosity in response to bisphosphonate treatment,(41) higher-powered HR-pQCT studies are needed to determine whether longitudinal changes in porosity are detectable.

Our results consistently indicated a greater trabecular and cortical bone response to alendronate in the weight-bearing distal tibia compared with the distal radius. This also was reflected in the µFEA estimates of bone strength, which were unchanged at 24 months at the tibia but significantly decreased at the radius compared with baseline. An important caveat of the µFEA method applied in this study is that it assumed fixed, homogeneous material properties. An advantage of this approach is that it allowed specific interrogation of the microarchitectural component of the biomechanical response to treatment independent of material effects. Accordingly, the scope of the analysis here was uniquely constrained to investigating geometric and microarchitectural effects related to ALN response. However, mineral compositional changes associated with antiresorptive therapies are thought to be a significant factor in their antifracture efficacy. Therefore, the µFEA approach applied in this study likely underestimates the true mechanical effects of alendronate.

A critical consideration in interpreting the longitudinal results of this study is the limited statistical power owing to an unexpectedly high attrition rate (∼50% for PBO). Retrospectively, power analysis indicated that 21 subjects per treatment group were required to detect a 5% difference in the change in Tb.vBMD, whereas only 13 PBO and 20 ALN subjects completed the second year. Because trabecular and cortical measures of bone microarchitecture are less precise than density, the lack of longitudinal and treatment-wise effects for these measures cannot be interpreted simply as a negative response. However, our longitudinal data for density, geometry, and microarchitectural changes in response to alendronate are remarkably consistent with the results of a recent 12-month multicenter HR-pQCT study in postmenopausal women treated with alendronate or denosumab.(22) Furthermore, it is possible, despite no significant difference between the final ALN and PBO groups in baseline age, body mass index (BMI), BMD, and biochemical markers of bone turnover, that the per-protocol analysis introduced some selective bias.

A novel component of this study was the use of regional analysis to provide anatomic localization of densitometric and structural changes. Because of substantial axial variability in density and bone morphology in these sites,(33,42) it is likely that certain regions provide greater sensitivity to treatment-related alterations or biomechanical stimuli. Indeed, while global measures of trabecular microarchitectural change did not reach statistical significance between treatments, specific cortical and trabecular regions (namely, the lateral and posterior quadrants of the radius and tibia, respectively) were found to show significant treatment effects in trabecular density and microarchitecture. The locations of these effects were consistent with regions of relatively low BV/TV and Tb.Th and are where the greatest age-related differences have been observed in a recent cross-sectional study.(33)

While the MANOVA analyses provide discriminatory information about treatment-induced alterations for independent measures of bone quality, they do not provide direct insight into how such alterations relate to functional efficacy. In general, the correlation analysis revealed that the biomechanical response to ALN treatment was primarily due to trabecular microarchitectural effects. In the radius, only trabecular bone measures were highly correlated with biomechanical response to ALN. In the tibia, in addition to trabecular structure, trabecular cross-sectional area (Tb.Ar) and porosity-related biomechanical deficits were negatively related to the overall biomechanical response to ALN. Changes in DXA aBMD were only moderately related to bone turnover changes and were relatively poorly associated with biomechanical response to ALN at both sites. These findings indicate that the response to ALN is primarily reflected by trabecular bone structure effects.

Interestingly, despite a trend toward a modest mean increase in trabecular number following 24 months of ALN treatment, the correlation between the percent change in Tb.N and the change in axial stiffness was found to be strongly *negative* for these subjects (Fig. 6). It is important to recognize that, on average, stiffness decreased significantly in the distal radius for the ALN-treated group. A possible explanation for this is that the anticatabolic action of ALN particularly inhibits resorptive fenestration of trabecular plates. In nonresponsive (or nonadherent) subjects with progressive early bone loss (osteopenia), trabecular fenestration may effectively increase the number of trabeculae through the conversion of a trabecular plate to multiple trabecular rods. This suggests that ALN responders in this early postmenopausal cohort maintained axial stiffness by preventing fenestration of trabecular plates. While this explanation may be consistent with early transient postmenopausal changes in bone structure (plate-to-rod conversion), it would not be expected to hold for elder osteoporotic cohorts, where bone loss is more typically characterized by loss off trabecular rods and increased spacing.(11) These observations highlight the complex nature of structure-function relationships and underscore the importance of investigating these interactions in a subject-specific manner.

The development of new therapeutic agents and the design of single or combination treatment strategies require an understanding the biologic factors that determine the treatment response. In general, baseline DXA aBMD measures were very poorly correlated with response in both the radius and the tibia. In contrast, baseline cortical geometry and density showed moderate to strong correlations with the biomechanical response in the tibia, whereas baseline cortical load fraction and porosity-related measures were significantly correlated with biomechanical response in the radius. Only Tb.vBMD and cortical porosity-related measures in the tibia correlated with the response in terms of bone turnover changes (BSAP only). This is consistent with a greater remodeling surface area available for bone formation following retardation of osteoclastic activity. Collectively, these results highlight the significance of cortical bone status in predicting response to ALN. Specifically, in this limited cohort, subjects with greater axial loads borne by the cortex, greater levels of intracortical porosity, and thicker cortices were found to be associated with a greater response to ALN.

There are several technical limitations to this study that should be acknowledged. First, common VOIs between baseline and follow-up scans are determined based on 2D periosteal cross-sectional area matching. This approach inherently assumes a constant cross-sectional area between measurements. A change in cross-sectional area via periosteal apposition would be expected to introduce an erroneous longitudinal offset between baseline and follow-up VOIs registered in this fashion. Recently, Boyd reported a pronounced structural variation along the distal-proximal axis of the radius in the distal region measured by HR-pQCT.(43) Collectively, this raises the possibility of specific biases in longitudinal changes determined with area-based registration. Full 3D image registration techniques, which have been shown previously to moderately improve short-term reproducibility of HR-pQCT measurements,(44) potentially would improve true region matching between longitudinal measurements. For this study, it is worth noting that clinical doses of alendronate in animal models have not been found to significantly increase periosteal apposition and therefore cross-sectional area.(45) Accordingly, malregistration owing to periosteal changes is not likely to be an important source of error.

A critical assumption of the standard analysis is a reliance on fixed-intensity thresholds and the derivation of trabecular BV/TV directly from Tb.vBMD. Alendronate has been found previously to modify tissue matrix mineralization by as much as 10%.(46) This has been shown to introduce moderate overestimation of changes in Ct.Th when calculated based on the cortical area and perimeter(47) and also would be expected to result in an overestimation of changes in densitometric trabecular BV/TV. As discussed previously, direct 3D quantification of cortical thickness was applied in this study in addition to the standard areal estimate and confirmed a true affect on tibial cortical geometry independent of matrix mineralization changes.

Recent phantom studies have found that Tb.vBMD measured by HR-pQCT is subject to geometric biases (ie, cortical thickness) likely related to beam-hardening and X-ray scatter.(48) While this is an important consideration for cross-sectional comparisons with large biologic variability, it is not expected to be a substantial source of error for measures of longitudinal change in an individual. Whereas the range of Ct.Th (0.5 to 3.5 mm) and Tb.vBMD (60 to 360 mg HA/cm^3^) considered by Sekhon and colleagues spanned broad cross-sectional extremes,(48) the longitudinal changes in Ct.Th and Tb.vBMD in this study were very small by comparison (<4%).

In this study we have characterized the microarchitectural, biomechanical, and bone turnover responses in early postmenopausal, women with low bone density to an established antiresorptive agent using noninvasive, high-resolution imaging. Significant treatment effects were detected for global density, but not microarchitectural properties. Changes in trabecular microarchitecture, not changes in cortical bone, were more highly correlated with ALN-related biomechanical response. However, the biomechanical response to ALN was significantly positively related to baseline cortical bone porosity in the radius and cortical geometry and density in the tibia rather than to baseline trabecular bone phenotypes. The dichotomous nature of the prediction of the therapeutic response and the actual manifestation of that response are important observations that could recommend and inform future clinical research directions significantly, including treatment customization and combination strategies that take into account cortical and trabecular structure phenotypes. In a hypothesis-generating study, these observations highlight the potential wealth of information high-resolution imaging techniques bring to clinical research.
